# Questioning assent: how are children's views included as families make decisions about clinical trials?

**DOI:** 10.1111/cch.12347

**Published:** 2016-05-02

**Authors:** L. Madden, V. Shilling, K. Woolfall, E. Sowden, R. L. Smyth, P. R. Williamson, B. Young

**Affiliations:** ^1^ c/o Institute of Psychology, Health and SocietyLiverpoolUK; ^2^ SHORE‐CUniversity of SussexBrightonUK; ^3^ Institute of Psychology, Health and SocietyUniversity of LiverpoolLiverpoolUK; ^4^ Institute of Child HealthUniversity College LondonLondonUK; ^5^ Department of BiostatisticsUniversity of LiverpoolLiverpoolUK

**Keywords:** assent, clinical trials, decision‐making, family, parents, semi‐structured interviews

## Abstract

**Background:**

Assent is used to take children's wishes into account when they are invited into clinical trials, but the concept has attracted considerable criticism. We investigated children's accounts of decision‐making with the aim of informing practice in supporting children when invited to join a clinical trial.

**Methods:**

We audio‐recorded qualitative, semi‐structured interviews with 22 children aged 8–16 years about being invited to take part in a clinical trial. Most children were interviewed with their parents. Analysis of the transcribed interviews examined the content of participants' accounts thematically, whilst also drawing on principles of discourse analysis, which examines how individuals use talk to achieve certain effects or social practices.

**Results:**

It was not possible to separate children's knowledge of the clinical trial, or their decision‐making processes from that of their parents, with parents taking a substantial mediating role in producing their children's decisions. Decision‐making gradually unfolded across time and events and was interwoven within the family context, rather than happening in one moment or in the clinical setting. Whilst children valued their parents' role, a case study of child–parent disagreement indicated how children can struggle to be heard.

**Conclusions:**

Decisions happen within a process of family dynamics, in contrast to ideas of assent that isolate it from this context. Parents have a substantial role in children's decisions, and thus how families come to provide consent. Reflecting this we argue that assent practices need to focus on supporting parents to support their children in learning and deliberating about trials. However, this needs to be accompanied by practitioners being alert to the possibility of divergence in child and parent views and enabling children's perspectives to be heard.

## Introduction

Whilst competent children can consent to clinical treatment, children entering clinical trials of medicines within the UK and many other countries are not legally permitted to give informed consent for themselves (Medicines for Human Use (Clinical Trials) Regulations 2004). This gives rise to the problem of how to include children in the research consent process. It is vital for children take part in clinical trials to ensure that advances in their treatments are evidence‐based; data on children's treatments cannot simply be extrapolated from clinical trials in adults (Gill 2002; Salazar 2003), as children's illnesses can be distinct to those of adults and children can respond to treatments in different ways compared to adults. Current practice is to seek proxy consent from an adult, usually the parent, and include the child's wishes by seeking their assent (Joffe *et al*. 2006; Alderson 2012). Assent is a problematic concept, as it must be applicable to a large range of children, from very young children who may struggle to articulate a view, to teenagers judged almost competent enough to give consent (Rossi *et al*. 2003).

Questions about how assent can be managed, achieved in practice and how much of a role a child can take in making the decision about whether to enter a trial are the topic of much debate (Alderson 2007; Baines 2011). It is not clear that assent gives the child any true legal power to dissent if parents want their child to enter a trial (Baines 2011; Blake *et al*. 2011). There are also suggestions that assent does not do justice to the extent to which children are able to participate in decision‐making (Alderson *et al*. 2006b; Snethen *et al*. 2006; Miller *et al*. 2008) and that it neglects the social context in which decisions are made. A child's engagement in decision‐making will depend on the relationships, processes and materials available to support them (Brody *et al*. 2005; Pinxten *et al*. 2008).

Much biomedical research on assent focuses on competence as a characteristic of the child (Tait *et al*. 2003; Hein *et al*. 2014) set apart from their context, investigating how far a child is able to achieve the building blocks of an agreement to take part in research. These components are taken to be, an understanding of the treatments, ability to deliberate and choose, to communicate an active preference and to understand their right to leave (Rossi *et al*. 2003; Miller *et al*. 2004). Some of this work aims to identify age‐related competencies (Rossi *et al*. 2003; Swartling *et al*. 2011) and inform decisions about the age at which children can meaningfully agree to participate in research.

In contrast, social science researchers have tended to view competence as context dependent, influenced by a child's experience of illness and relationships with parents over the course of decision‐making (Alderson *et al*. 2006b). Linked to this emphasis on the context and relationships, decision‐making about children's participation in research has been conceptualized as a ‘family consent’ process (Miller *et al*. 2004; Gibson *et al*. 2011), which draws attention to the role of family structures and hierarchies in decision‐making (Heritage & Maynard 2006). Traditionally, parents have responsibility for making decisions on behalf of children and might therefore be assumed to approach decision‐making from a position of authority. However, parents may feel vulnerable as they balance their authority with expectations to involve their child in decision‐making (Silverman 1987; Arribas‐Ayllon *et al*. 2011; Nuffield Council on Bioethics 2015). Fears of making the ‘wrong’ decision and the regret this would bring, and about children's susceptibility to potentially harmful treatments (Read *et al*. 2009; Shilling & Young 2009; Salmon *et al*. 2012), may exacerbate parents' sense of vulnerability.

Whilst much has been written about assent as a concept, little empirical work has involved children to inform these ideas and whether they resemble children's decision‐making in practice. Empirical work is needed to inform conceptualizations of assent, identify how best to support children in making decisions about research participation and inform the design of resources to support their decisions. Much of the empirical work that is available sidesteps the contextual complexities by using hypothetical scenarios, or involving children and families who have no experience of trials (Corrigan 2003; Miller *et al*. 2004; Hunfeld & Passchier 2012) and evidence is needed about decision‐making in real world situations. Inductive qualitative methods are helpful in exploring taken for granted assumptions and processes that may be otherwise be overlooked, particularly in areas that have previously been little investigated (Murphy *et al*. 1998). We adopted such an approach, interviewing children who had been invited to join a trial and exploring how they and their parents described this invitation and the decision‐making process. We considered how well assent, as it is conceptualized in both the biomedical and social science literature, is compatible with how families make decisions about children's entry into clinical trials. Our overall aim was to inform practice in supporting children's decision‐making about research.

## Methods

### Sampling and recruitment of clinical trials and families

We interviewed children as part of a larger qualitative study (Shilling *et al*. 2011b; Shilling *et al*. 2011a), called RECRUIT, investigating recruitment to four publically funded placebo‐controlled, randomized clinical trials of medicines for children. Recruitment to these trials involved one or more face‐to‐face discussions between families and clinical trial clinicians. Whilst the children's interviews are the focus of this paper, the RECRUIT study also investigated parent‐practitioner communication during recruitment. The wider dataset comprised audio‐recordings of discussions between families and family‐clinicians about the trials, as well as interviews with parents and clinicians. The methods of this larger study are described in detail elsewhere (Shilling *et al*. 2011b).

From a pool of 14 potentially eligible trials, we purposively sampled four trials to encompass variation in the severity of illness and circumstances under which families were invited to join the trials. Clinical trial clinicians at 11 sites invited families to participate in RECRUIT, usually in person or by telephone. RECRUIT interviewers subsequently telephoned interested families to give more information about the study and arrange the interviews. Sampling across the four trials comprised a mix of consecutive and purposive sampling. Consecutive sampling aimed to avoid clinicians ‘cherry picking’ families, whilst purposive sampling aimed to encompass diversity in socio‐demographic characteristics and whether children participated in the trials, declined, withdrew or were ineligible. Of the 95 families invited for an audio‐recording of the clinician–parent discussion and/or qualitative interview, members of 60 (63%) families were interviewed; the remainder were either not approached for interview following the audio‐recorded discussion (*n* = 5) or did not consent to interview. Within the three trials that are the focus of this paper (the fourth trial focussed solely on neonates and is not discussed further) we interviewed members of 48 families. Of these, 22 families were from the most materially deprived fifth of the UK population (based on the Index of Multiple Deprivation) and three families were from a minority ethnic group.

As the topic of clinical trial recruitment was rather abstract and likely to be difficult for young children, we did not interview those aged less than 8 years. Out of the remaining 34 families with children aged eight or over, children from 12 families were not interviewed, either because parents advised that an interview would not be suitable (the children had severe neurodevelopmental problems), or because the child declined. Of the 22 interviewed children, 11 were female; eight were aged 8–10 years, eight were 11–13 years and six were 14–16 years. Eighteen interviewed children lived in North West England or the West Midlands, and four in Northern Ireland. Thirteen children were interviewed about a rheumatology trial, seven about a respiratory trial and two about a neurodevelopmental trial. Most interviews took place in the family home and lasted between 15 and 30 minutes. Eight children asked to be interviewed jointly with their parents, two were interviewed alone and twelve parents (11 mothers, 1 father) were present for at least some of the child's interview and contributed to some degree. One child's interview did not contain any content relevant to the trial and was therefore excluded from the analysis.

### Ethics

Our access to children was usually negotiated via their parents, and reflecting this we obtained informed consent for children's participation from their parents. After discussing the study with children, if researchers thought children understood the study they sought their informed consent (in addition to parental consent) and asked children to sign their own consent forms. Otherwise researchers sought children's agreement to participate and asked them to sign an assent form. A UK National Health Service research ethics committee gave approval for RECRUIT (Northwest 5 Research Ethics Committee: 07/MRE08/6). All data have been anonymized, and specific details of the trials, as well as exact ages of the children omitted to minimize risk of participants being identified.

### The trials and recruit interview schedule and procedures

Of the three trials, one investigated treatment for a symptom of a chronic respiratory illness, one investigated treatment to combat a major side effect of treatment for a rheumatological illness and one investigated a treatment to manage ongoing problems associated with a neurodevelopmental condition. During the face‐to‐face discussions with families, clinicians outlined the trial aims, main procedures, what participating would entail, treatments being investigated and any accompanying risks. Information leaflets containing similar details about each of the trials were provided to both parents and children; the latter were available in several age‐appropriate versions. As we describe elsewhere (Shilling *et al*. 2011b), children and parents were often largely silent in the discussions with clinicians, although they usually had several weeks afterwards to make a decision about the trial. During this time families could consult the leaflets, as well as contact clinicians with queries, although we do not know how frequently families initiated such contact.

VS and ES conducted topic‐guided semi‐structured interviews with children between March 2008 and January 2010, in the families' homes. They used several techniques to help children, particularly those in younger age groups to feel comfortable. For example, at the start of the interviews the researchers laid out cards with familiar pictures (such as a horse or boat), on one side and a question on the other side. Children selected a card to begin a topic. After each topic had been discussed they placed a sticker on the card to mark it as complete before selecting a new card. This aimed to help children feel comfortable by bringing a game‐like quality to the interaction. If parents were present, interviewers were careful to direct their questions towards children to maintain a sense that the child was the ‘owner’ of the interview. We developed different versions of the topic guides for younger and older children. These explored the background of the child's illness, their views of the trial, how well their needs were met, how they had made the decision about the trial and who influenced it, the written information provided and their views on clinical research. Example questions included: ‘If I ask you to think about [trial] now, is there anything that comes to mind? Did you have any worries about taking part in the [trial]? Were you able to ask questions?’ Researchers aimed to cover the same topics in each interview, whilst adapting questions for each participant and prompting as appropriate so that interviews were conversational. We audio‐recorded all interviews, and transcribed them verbatim, with some idealization such as adding punctuation.

### Data analysis

LM led the analysis with support from BY and VS. Analysis explored the content of participants' accounts inductively and thematically, whilst also drawing on the principles of discourse analysis (Potter & Wetherall 1987; Silverman 1987). Discourse analysis focuses on the ways that participants talk, treating interviews not as presentations of participants' perspectives, but as acts to achieve particular effects, such as influencing or eliciting certain responses from others (Austin 1962). Therefore, we considered participants' accounts in the context of imperatives for them to speak in socially and morally expected ways. For patients and parents describing their decision‐making there is pressure to present themselves as responsible and active deciders (Silverman 1987; Arribas‐Ayllon *et al*. 2008). We attended to participants' work of creating this effect, particularly in parts of the interviews where children and parents were speaking to each other and explaining themselves interactionally. Procedurally the analysis was iterative, with transcripts being read several times, first to identify material relevant to the research questions, before analysing this material in detail to consider how participants positioned themselves in relation to the trial information and the different ways in which participants talked about the decision‐making, and the discursive strategies children and parents used to negotiate their positions. LM and BY periodically discussed the developing analysis and all authors, particularly VS, contributed to this process by commenting on reports of the analysis containing extensive data extracts.

We addressed quality by considering ‘negative’ cases, as illustrated in the case study that we describe (Murphy *et al*. 1998), and by providing contextual detail to help readers make sense of the findings and assess their transferability. More broadly, we considered the analysis in terms of its catalytic validity, that is, its potential to inform practice (Kincheloe & Mclaren 2000). Excerpts quoted (Table 1) are representative of categories found in the analysis of data as a whole; all names are pseudonyms and children's age ranges are indicated. Where quotes have been shortened for brevity or to remove potentially identifying detail, omitted text is marked with ‘[…]’.

**Table 1:** The work of understanding trials and parents' role in producing assent

**Extract 1**: *14–16 years*
*Ellie*: *the leaflets have to have them*, *although I don*'*t read them*, *but* […] *my mum does and* […] *and she goes through it but kids don*'*t really*. *Well I*'*m saying I don*'*t read*… *I don*'*t like reading them*.
**Extract 2**: *8–10 years*
*Researcher*: *Do you prefer to chat to the doctor or chat to mum*?
*Jack*: *Just watch TV or play on my DS or look at magazines*.
**Extract 3**: *8–10 years*
*Jordan*: *Yea*, *I talk to my mummy and I don*'*t know what you said*, *I can*'*t remember*.
*Mother*: *You had a leaflet*, *didn*'*t you*, *and you read that and I just told you a bit about it*, *and then*
*Jordan*: *That was it*.
*Mother*: *Asked you what you thought*
**Extract 4**: *14–16 years*
*Lisa*: *Yeah*, *the whole idea that other people had been doing it and that it*'*s*, *it*'*s not a new drug because mum was saying I would have* […] *I probably wouldn*'*t have been as open to it if I hadn*'*t been on a similar medicine before*.
**Extract 5**: *11–13 years*
*Mark*: *I didn*'*t want to take part in the trial at first*, *in case there were injections*. *Then mother explained there wouldn*'*t be*. *So I was happy to do the trial*.
**Extract 6**: *8–10 years*
*Researcher*: *Can you remember who it was who first mentioned the trial*?
*Emily*: *Um*, *not it wasn*'*t*, *was it*, *mum*?
*Mother*: *The very first person that mentioned the study was* [*consultant name*]
**Extract 7**: *8–10 years*
*Researcher*: *Did you feel like you were free to leave the trial*?
*Gemma*: *I don*'*t know*. *Can you help me mum*?
*Mother*: *I don*'*t know how you felt babe*! *Did you ever think* ‘*Oh*, *I don*'*t want to do this no more*’? *With your fizzy medicine and you*'*re*..?
*Gemma*: *Not really*, *no*
*Mother*: *Well there you go then*, *babe*.
**Extract 8** †: *14–16 years*
*Ellie*: *I like my mum being in on it so that if there*'*s anything I*'*m concerned about I can say to my mum*
**Extract 9** †: *11–13 years*
*Oliver*: *If I said* ‘*yes*’ *but my mum and dad said didn*'*t approve well*. *I just probably would say* ‘*can we talk about it*?’ *because if they don*'*t approve I won*'*t be*, *feel confident about it will I*?

† These extracts are reproduced from Shilling *et al*. (2011b) Processes in recruitment to randomized controlled trials of medicines for children (RECRUIT): a qualitative study. *Health Technology Assessment*, 15, 1–116.

## Results

### Overview

In the following sections we describe how children's understanding of the trials arose from collaborative work within families. We particularly focus on one child whose parents overtly persuaded her to enter a trial, yet our findings also show other parents being similarly, if more subtly, influential in shaping their child's engagement with the trial. The findings show that children not only evoked such responses from parents, but also that children relied upon and mostly valued this work by parents. Quoted data extracts are shown in Tables 1 and 2.

**Table 2:** A case study: entering a trial then leaving it

**Extract 10**: *11–13*, *female*
*Katy*: *I felt a bit under pressure*, *yea*! *Umm*. *I*'*m not sure because I don*'*t think it was my decision*, *I think it was Mum*'*s and Dad*'*s really*! […] *But*, *Um*, *I just decided to do it in the end*, *to kind of get them to be quiet*!
**Extract 11**: *Mother*: *We did persuade Katy*, *I suppose when the study started*, *she wasn*'*t normal Katy*, *you know*. *She would*, *I suppose*, *agree to lots of things maybe*. *And then as it got towards early summer*, *you know*, *she*'*s very much getting her own voice back*. *And of course*, *she was wheedling away in*, *looking every way possible to try and stop taking different medicines* […] *it*'*s not like you*'*ve got an eight year old and* ‘*you have to take that*!’. *She knew it was optional because she*'*d been told that in the first place*. *Well if she hadn*'*t been told it was optional we might have managed to keep her on*!
**Extract 12**: *Mother*: *Honestly*, *she can be quite strong willed when she*'*s getting better and she*'*s been through such a lot* […] *as she*'*s going to get better*, *Katy will say no to lots of things*.

### The work of understanding trials

From their accounts of being invited to join the trial, it was apparent that some children were content to delegate much of the work of finding out about the trial to their parents. As extracts 1 and 2 illustrate (Table 1), these children implied that they wanted little engagement with the written or verbal information about the trials, instead relying on their parents to do this work. This is despite children having been provided with adapted versions of the trial information leaflets, and clinicians' having tried to direct explanations and questions to children during the family‐clinician discussions about the trials.

Other children had engaged with the trial information to some degree, but it was still common for them to describe how their knowledge or opinions of the trials had derived from their parents' explanations. Extract 3 (Table 1) shows how Jordan, a younger child, relied on his mother to provide the bulk of the description for the interviewer. As the mother's talk indicates, she not only mediated information about the trial, but also the process of eliciting her child's views. Similarly, in extract 4 (Table 1), whilst Lisa offered a sophisticated view about making the decision to join a trial, her phrase ‘because mum was saying’, indicated that parts of Lisa's description had come from her mother. Moreover, in using this phrase, Lisa could be seen as invoking adult views in order to add legitimacy to her account. Several children went further and explicitly described how their parents went through a process of explaining the trial to them. In extract 5 (Table 1), Mark spoke of having initially misunderstood the trial treatment. He then referred to his mother's explanation of the treatment and how this had helped him to move forward in his understanding of the trial and give assent.

The work of understanding the trials could therefore be seen as located not within the child themselves, but as a relational process, in which children and parents collaborated to varying degrees, but with parents taking a substantial mediating role (Corrigan 2003).

### Parents' role in producing assent

The kind of collaboration between parent and child as seen above was not only reported by children; it could be seen in how participants talked within the interviews. As with Lisa above, children's reports of their parent's perspective could help them achieve a legitimacy that they might not have had alone. Children also frequently requested help from parents as in extract 3. The contribution of parents varied in its impact on the interview. In extract 6 (Table 1), for example, Emily seemed to struggle with a somewhat technical question, which her mother stepped in to answer after Emily had asked for her help. However, parents' contributions could go beyond simply helping. As extract 7 (Table 1) indicates, parents could subtly yet significantly transform the discussion, in this case by reworking the interviewer's question so that Gemma ended up answering a much ‘weaker’ question, which lacked the enquiry into voluntarism of the original question.

Therefore, parental input, although collaborative, did not just involve neutrally helping their children with interview questions. Parents' interjections inevitably reflect their own views and priorities, and through this type of collaborative work, parents directed their children's understanding of the trials. As extracts 8 and 9 (Table 1) illustrate, children spoke of relying upon and valuing their parents' collaboration.

These collaborative styles of interaction were a recurrent pattern throughout the data and taken together suggest that it is not realistic to treat the child's preferences and opinions as separate to those of their parents. It is not only that parents explicitly influenced children's views, with several children reporting having changed their minds over the course of the decision‐making process; parents could be seen as working in nuanced ways to ‘produce’ assent by directing their child's engagement with the trial, comprehension of procedures and ability to show a preference. The extent of the parental role adds to doubts about assent and whether it is a meaningful concept (Baines 2011).

## A case study: entering a trial and then leaving it

The first part of this paper has considered some specific elements of how children's preferences are developed and managed within the family. We have looked at how the child's understanding is based on the explanations of parents, and how parents can also subtly reframe issues to mediate their child's account of the trial. This strong role of parents can erode the notion that assent is the child's own view, as parents have considerable, and often subtle persuasive power, in how they develop their child's knowledge and engagement with the trial.

In the final section we consider the case of one family in more detail, to give a sense of the entire decision‐making process, and how it unfolded over several months. This also aims to illustrate how we worked through the data analysis, to incorporate ‘negative’, or less simple, cases (Murphy *et al*. 1998). We particularly describe how this family's account of decision making was embedded in day‐to‐day family life (Arribas‐Ayllon *et al*. 2011), and intertwined with coming to terms with a diagnosis of a serious illness.

### Doing some good: entering the trial

Shortly after Katy's diagnosis she was invited to enter a clinical trial for a medicine that offered the potential to minimize a long term side effect of one of the standard medications for her condition. She initially entered the trial, with the key argument from her parents, that the trial treatments would do no harm, and might do some good in offering a potentially protective treatment and a level of monitoring that Katy was not receiving as part of her standard care. Whilst Katy initially entered the trial based on her parents' views, extract 10 (Table 2) shows there was already some disagreement within the family at the point of trial entry. This was described with ambivalence about who made the decision. Although Katy had relinquished her own claim to voluntarism, by describing it as her parents' decision, she explained the process in negative terms, referring to ‘pressure’, and wanting her parents ‘to be quiet’.

### Too many treatments: experience of the trial

Whilst Katy's mother described her daughter's position as ‘*Poor Katy just wanted to be left alone*, *didn*'*t you*?’ in extract 11 (Table 2) several different positions were introduced about the significance of Katy's own view. The parents framed themselves as using ‘persuasion’, whilst in contrast describing Katy as ‘wheedling’, and looking for ways to minimize the number of treatments in her regimen. Her mother indicated several circumstances that strengthened Katy's position to make a decision for herself. These included Katy's age, and the implications that follow for voluntarism, Katy ‘getting her own voice back’ as she recovered from the illness, and being empowered by the trial's assent process. Katy's mother indicated she would have liked to keep her daughter in the trial despite Katy's desire to leave. She also minimized Katy's reasons for wanting to leave the trial and framed the assent process as a rather negative influence in giving Katy the tools to leave.

### ‘Strong will’ and ‘persuasion’: the management of a decision to leave

In her final summary, Katy's mother described the decision to withdraw from the trial as one that Katy largely made. Katy's withdrawal was the conclusion of disagreements that took place over a series of months and were managed within the family. Katy gained a form of authority within this family process through her ‘strong will’, from having ‘been through such a lot’, and also from her reluctance to enter the trial from the beginning and maintaining this stance over several months. Katy being of an age that meant she could offer some deliberation and must have some of her own say added to her authority. This package of circumstances gave Katy a stronger relationship to the deliberations required of an informed consent model. However, her dissent was not attained in any clear cut way. Indeed, she might never have achieved this final triumph of her views without the assent process that informed her of her right to leave, and she still had a lengthy struggle, over time and through the dynamics of the family, to finally achieve it.

## Discussion

We explored how decisions about consent for children's clinical trial enrolment are managed in the context where these decisions are made. We found that children's views owe much to the explanations and subtle scaffolding work of parents. Children were strongly influenced by their parents and seemed to want this influence. Even when a child held strong views about a particular treatment, these often changed readily in discussion with a parent.

We looked in more detail at the case of one child, Katy, and her parents. We saw how the direction of decision‐making changed several times, with disagreements being managed within the family, rather than within the assent process offered by the clinical team. These decisions unfolded by degrees over a long series of events (Rapley 2008), with the child's eventual authority embedded in her ongoing experience of illness (Alderson *et al*. 2006a). The decisions were not easily located in one individual, rather they were distributed over several individuals (Ashcroft *et al*. 2003; Edwards & Edwards 2012) and turned on many issues that were neither clinical nor ethics‐related, such as the child's wish to get her parents ‘to be quiet’, and to cut down on treatments that she disliked (Corrigan 2003). What we saw that this family's practices were much more persuasive, and some might argue, coercive, than those found in studies of children's and parents' general views about medical research or in hypothetical trials (Rossi *et al*. 2003; Swartling *et al*. 2011), where parents were reported to support children in making decisions, without substantially influencing them or dismissing dissent.

Our findings suggest that even the oldest children rely heavily on their parents to translate and make sense of information. Parents played such a substantial role in understanding what a trial is, as well as the reasons for and against joining, that it is hard to see how a child could dissent completely independently from their parents. They subtly shaped their child's engagement with the trial and added more overt persuasion or pressure at many points to the extent that parents could be seen as ‘producing’ assent. This is in contrast to the some of the biomedical literature, which sees children's decisions as separable from their parents and treats assent as if it can be achieved relatively independently of the family context (Tait *et al*. 2003; Hein *et al*. 2014). It has been argued that such approaches misunderstand assent (Sibley *et al*. 2012), and that assent should be treated simply as a process to involve children in the decision‐making, in whatever way is appropriate for an individual child (Nuffield Council on Bioethics 2015). The latter approach to assent emphasizes the needs and preferences of each particular child in the context of their family, and how s/he can be supported and engaged in the decision‐making process. The findings we report are largely consistent with this notion of assent, but also point to the considerable role of the parents in decision‐making (Miller *et al*. 2004; Joffe *et al*. 2006).

### Limitations

This study has several limitations. First, it included three trials for chronic conditions, where the process of being informed about the trials, invited and seeking assent tends to happen over a long period. Therefore, elements of our findings may not be transferable to clinical trials conducted in more time constrained contexts. Second, parents in our study were highly engaged with informing their children and discussing the trial with them. It is possible that parents who took part in this study are committed to mediating for their child in these ways, and our findings may not be transferable to other families. Third, in all except two cases, parents were present for at least part of the child's interview. Children could readily turn to parents for help and parents could readily interject, potentially leading to an amplified picture of parents' role. Nevertheless, this context mirrors how discussions about clinical trials take place in practice. Finally, there are several outstanding questions about assent requiring specialist ethical analysis that are beyond the scope of this empirical paper. The Nuffield Council has recently produced such an ethical analysis (Nuffield Council on Bioethics 2015).

### Practice recommendations and suggestions for further research

This paper demonstrates the substantial role of parents in the assent process. Based on this, we suggest that practitioners and information resources need to a focus on supporting parents to support their children's engagement in trial decision‐making. Such support for parents would be consistent with what we observed of their role. It would also reflect how children valued and relied upon the input of their parents, and in these particular trials, how much decisional work was done within the family and outside the clinic. That is not to say that practitioners and resources should only engage with children via their parents – clearly the principles of involving children and promoting their trust requires practitioners to engage with children directly. The case of parent–child disagreement that we saw, where the parents persuaded their reluctant daughter to enter a trial (only for her to subsequently withdraw), also reminds us that parental support alone is not sufficient. We therefore suggest that alongside supporting parents to support their children, practitioners should look out for diverging views between children and parents, and offer children the chance to discuss the trial separately from their parents. Further research should identify and evaluate the role of practitioners in delivering this more personalized and family orientated approach to consent, and particularly, to understand how practitioners can support parents to support their child's engagement outside the clinic or hospital, whilst also enabling children's views to be heard.

**Key messages**

- Most of the relatively small body of research on children's accounts of assent to clinical trials does not take account of the family and social context or involve participants with real experience of trials. This study aimed to address these limitations and inform practice in children's assent.
- We found parents had a considerable mediating role in how children understood trials and had a large influence on their views. This role was one that children largely seemed to welcome.
- Decision‐making was embedded in family dynamics and relationships and happened over time as events unfolded in the management of the child's illness.
- In recognition of how decision‐making is embedded within the family and children's reliance on parents, practitioners should consider how to support parents to support their children in understanding and deliberating about clinical trials.
- This should be accompanied by efforts to identify divergences of opinion between the two parties and to enable children's perspectives to be heard, perhaps by offering children the opportunity to discuss clinical trials separately from their parents.
